# Spatiotemporal analysis of hospital admissions for primary care-sensitive conditions in women and children in the first 1000 days of life

**DOI:** 10.1371/journal.pone.0269548

**Published:** 2022-06-09

**Authors:** Isabelle Aguiar Prado, Núbia Cristina da Silva Rocha, Thiago Augusto Hernandes Rocha, Erika Bárbara Abreu Fonseca Thomaz

**Affiliations:** 1 Departamento de Saúde Pública, Programa de Pós-graduação em Saúde Coletiva, Universidade Federal do Maranhão, São Luís, Maranhão, Brazil; 2 M.A.T.H. CONSORTIUM – Methods, Analytics, and Technology for Health, Belo Horizonte, Minas Gerais, Brazil; 3 Duke Global Health Institute, Duke University, Durham, North Carolina, United States of America; The Ohio State University College of Medicine, UNITED STATES

## Abstract

**Objective:**

To analyze the spatiotemporal distribution of hospital admission rates for primary care-sensitive conditions (PCSC) in women and children in the first 1000 days of life in Brazil.

**Methods:**

Ecological study, with spatiotemporal analyses, using secondary data from Brazilian municipalities. PCSC in women, related to prenatal care and childbirth, and in children under two years old, from 2008 to 2019 were used to characterize trends and formations of spatiotemporal clusters/outliers. Crude PCSC rates were calculated and adjusted by the local empirical Bayesian method, presented in choropleth maps. We also used Anselin Local Moran I type analyses to identify spatial clusters, and space-time cube with clustering by emerging hotspot, followed by time series clustering, for analysis of spatiotemporal trends (alpha = 5%).

**Results:**

A total of 1,850,776 PCSC were registered in pregnant women, puerperae, and children under two years of age in Brazil, representing 1.7% of the total number of hospital admissions in the period. PCSC rates showed different behaviors when the groups of women and children were evaluated, with a predominant growing trend of 109% in admissions in the first group and a reduction of 34.4% in the second. The North, Northeast, and Midwest regions had larger high-risk clusters and more significant increasing trends in PCSC in the two subpopulations studied.

**Conclusions:**

Health actions and services in primary care may be reducing hospital admissions for children, but they are not being effective in reducing hospital admissions for women for causes related to prenatal care and childbirth, especially in the North, Northeast, and Midwest of Brazil. Investments in the qualification of care over the thousand days are urgent in the country.

## Introduction

Hospital admissions due to primary care-sensitive conditions (PCSC) are an indicator of primary health care (PHC) effectiveness. This indicator includes a set of health problems that could be reduced/avoided from the effective action of PHC (with strategies such as disease prevention, diagnosis, and early treatment of acute pathologies, and control and monitoring of chronic pathologies), avoiding hospitalizations [1, 2].

Previous Brazilian studies point to the declining trend of PCSC in children under five years of age in different Federative Units, such as Ceará, with a reduction of 65% (2000–2012) [3]; Espírito Santo, 28.79% (2000–2014) [4]; Pernambuco, 42.8% (1999–2009) [5]; and Piauí, 17% (2000–2010) [6]. These studies associate lower PCSC with the expanded PHC coverage [4–6] and other public policies, such as the *Bolsa Família* (Family Aid), a conditional cash transfer program [3].

However, when evaluating the component related to childbirth and postpartum, the decline in PCSC rates was negligible in women, with minimal reductions, 0.1% from 2003 to 2012 in Minas Gerais [7], and even an increase of 0.6%, from 2008 to 2012, in Pernambuco [8]. These data reflect the global setting of maternal health, without effective responses compared to other priority areas [9].

A systematic review conducted by Rosano et al. (2012) concluded that access to PHC is inversely related to the number of admissions due to preventable conditions [10]. Thus, high PCSC rates in a population (or its subgroups) may indicate problems accessing the health system or its performance [11].

Considering that the first 1000 days—from conception to the second year of life—are a window of opportunity for health intervention, in which emerging problems can adversely impact the child’s growth and development [12, 13], it is crucial to understand the PHC behavior in offering care to this population. However, we did not identify nationwide studies of PCSC in women and children in the first 1000 days, considering more current periods, with spatiotemporal analyses.

Despite the general improvement in maternal and child indicators over the last two decades in Brazil, we hypothesize that significant regional differences persist in the distribution of PCSC rates in the first thousand days in the country, which can be identified from an analysis that considers the spatial component, identifying critical regions. Thus, this work aims to analyze the spatiotemporal clustering patterns of PCSC in women and children in the first 1000 days of life in Brazil in the 2008–2019 period.

## Methods

### Study type and location

This ecological, longitudinal, and analytical study employed secondary databases, considering the 2008–2019 period and the Brazilian municipalities were the units of analysis.

Brazil is the largest country in Latin America, with approximately 212 million inhabitants and 8,514,876 km^2^ of land area. It has 5,570 municipalities, distributed into 27 Federative Units and five geopolitical regions (North, Northeast, Southeast, South, and Midwest) [14]. It is a middle-income country, with an average Human Development Index (HDI) of 0.754 and marked regional differences, which are also expressed when assessing health indicators and service coverage [14].

### Study population

The study population consisted of all cases of PCSC in pregnant women/puerperae and children up to two years old, registered in the Hospital Information System (SIH), from 2008 to 2019. We considered the Brazilian list of conditions of the Brazilian Ministry of Health for defining PCSC [11]. Admission data with inconsistent or incomplete information and births (ICD-10: O80 to O84)—as they represent a natural outcome—were excluded.

### Data sources and study variables

All data analyzed was obtained from public secondary databases, and no approval of Ethics Committee was necessary.

PCSC data were collected from the SIH, SUS Department of Information and Informatics (DATASUS), considering the place of residence, from 2008 to 2019. The onset of the study period was defined from the implementation of the SUS Table for Procedures, Medicines, Orthotics, Prostheses, and Special Materials from January 2008 [15].

The PCSC rate for women and children was calculated separately and jointly to assess PCSC in the first thousand days. For children, we included all hospital admissions for sensitive conditions in the Brazilian list, considering data referring to age group and identification of the chapter of the International Code of Diseases (ICD-10). Regarding PCSC in women, were included all the Brazilian admissions in the group of sensitive conditions related to prenatal and childbirth care directly affecting this group (Infection of Genitourinary Tract in Pregnancy—ICD O23). Hospital admission records with missing CID identification data, city/state of residence or age were excluded.

The populations considered in the denominators to determine PCSC rates per year studied were: a) for women, the number of live births (LB) and stillbirths (SB) per year, in the absence of reliable information on the number of pregnant women, parturients, and puerperae in the territory; and b) for children, the population under two years of age. Information on the number of LB and SB by municipality was collected from the Live Births Information System (SINASC) and the Mortality Information System (SIM). The population of children was obtained from Brazilian Institute of Geography and Statistics (*Instituto Brasileiro de Geografia e Estatística*–IBGE) data, according to the 2010 Census and inter-census projections.

### Data analysis

The PCSC spatiotemporal clustering patterns were analyzed in three stages. The first stage evaluated the spatial distribution of PCSC in the first thousand days, considering the municipalities of residence. The second stage aimed to identify spatiotemporal clusters by PCSC rates in women and children under two years. In the last stage, clusters were identified by the trend of the evaluated indicators.

Statistical analyses were performed using Stata, version 14.0 (StataCorp, Texas, USA) and ArcGis Pro software, version 2.7 (Esri Inc, California, USA), considering a significance level of 0.05. The choropleth maps were prepared using the QGIS software, version 3.12.0 (QGIS project), using the IBGE cartographic grid of the Brazilian municipalities (SIRGAS 2000 Geographical Coordinate System).

#### First stage—Spatial distribution analysis

The subjects were georeferenced based on their municipality of residence to characterize the spatial distribution patterns of PCSC in the study groups. For descriptive analysis, data were presented in quadrennia (2008–2011, 2012–2015, 2016–2019), assessing the event’s spatial distribution over the period evaluated.

The crude PCSC rates were calculated considering the total number of PCSC in women (due to diseases related to prenatal care and childbirth) and in children under two years old, divided by the population of the municipality in the period (LB and SB, to calculate PCSC in pregnant women/puerperae; population < 2 years to calculate PCSC in children), multiplied by 10,000. Then, we calculated the PCSC rates adjusted by the local empirical Bayesian method to evaluate the neighbor relationships, controlling for random fluctuations [16]. The rates were presented on choropleth maps, considering the municipalities as the unit of analysis.

#### Second stage—Spatiotemporal clusters and hotspot analysis

We adopted the Anselin Local Moran’s I analysis to identify PCSC spatial clusters over the thousand days, weighted by the temporal component, from which the municipalities were classified into clusters of the following types: a) High-risk cluster (High-High autocorrelation pattern)—includes municipalities with high PCSC rates surrounded by other municipalities with high rates; b) Low-risk cluster (Low-Low autocorrelation pattern)—includes municipalities with low PCSC rates surrounded by other municipalities with low rates; c) Low-High Outlier—municipalities with low PCSC rates surrounded by those with high PCSC rates; d) High-Low outlier, municipalities with high PCSC rates surrounded by those with high rates (High-Low autocorrelation pattern) [17].

A space-time cube (STC) of the rates was employed to assess the spatiotemporal distribution of PCSC rates in women and children, considering Brazilian municipalities as the unit of analysis and a time interval of one year. Then, we conducted the emerging hotspot analysis, which identifies the p-value and z-score of each location in the cube, categorizing the municipalities into hotspots, with a tendency to grow over the period studied; cold spots, with a tendency to decrease; or no pattern detected [18].

#### Third stage—The spatiotemporal trend

Then, we built clusters according to the temporal trend of PCSC rates in the municipalities using the Time Series Clustering tool [19], ArcGis Pro software. This analysis clusters STC locations by similarity of the temporal trend throughout the studied period. The clusters were created considering the “correlation” characteristic, which groups time series that tend to remain in the same proportion among themselves, increasing or decreasing their value.

Additionally, this analysis provides graphs showing the mean and median rates (medoids) of the clusters formed in each year evaluated, allowing an evaluation of the general mean/median of the series in each cluster.

## Results

In Brazil, 353,119 PCSC were registered in women for prenatal care and childbirth conditions, with 1,497,657 hospital admissions in children under two years of age, from 2008 to 2019, representing 1.7% of the total number of admissions in the period. The mean PCSC rate in women due to prenatal care and childbirth conditions increased by 109% over the period studied, while the mean PCSC rate in children under two years of age decreased by 34.4% (Table 1).

**Table 1:** PCSC rate in pregnant women, puerperae, and children under two years of age in Brazil and macro-regions.

	2008	2009	2010	2011	2012	2013	2014	2015	2016	2017	2018	2019
	Mean (±sd)	Mean (±sd)	Mean (±sd)	Mean (±sd)	Mean (±sd)	Mean (±sd)	Mean (±sd)	Mean (±sd)	Mean (±sd)	Mean (±sd)	Mean (±sd)	Mean (±sd)
**PREGNANT WOMEN/ PUERPERAE**												
Brazil	52.90 (±145.28)	61.35 (±163.14)	70.14 (±193.72)	74.99 (±192.45)	83.53 (±196.68)	86.40 (±194.95)	93.34 (±202.57)	96.76 (±198.17)	96.00 (±209.36)	98.21 (±216.12)	110.65 (±237.56)	110.56 (±225.50)
North	74.68 (±137.75)	96.65 (±166.53)	116.62 (±332.57)	123.61 (±301.03)	124.35 (±254.98)	159.24 (±326.18)	186.03 (±354.83)	176.62 (±337.07)	173.35 (±317.35)	204.11 (±346.88)	226.15 (±388.08)	225.19 (±346.82)
Northeast	15.95 (±48.59)	19.31 (±57.05)	25.34 (±88.3)	28.95 (±82.87)	35.07 (±106.13)	34.63 (±97.05)	42.94 (±106.0)	48.54 (±110.77)	57.00 (±133.68)	58.52 (±135.21)	70.2 (±148.52)	76.16 (±175.09)
Southeast	68.99 (±173.81)	83.42 (±179.28)	96.73 (±209.93)	102.95 (±202.52)	105.10 (±183.17)	98.53 (±173.01)	113.08 (±191.46)	109.57 (±185.0)	104.26 (±199.68)	103.44 (±213.29)	113.52 (±237.9)	110.95 (±212.46)
South	73.62 (±182.46)	74.36 (±215.3)	77.51 (±194.9)	76.3 (±200.55)	103.79 (±245.93)	107.95 (±241.54)	108.16 (±235.03)	112.51 (±213.23)	110.78 (±249.78)	107.12 (±231.52)	119.36 (±257.35)	108.93 (±215.24)
Midwest	63.67 (±154.79)	76.96 (±187.6)	83.91 (±211.39)	101.95 (±256.55)	101.923 (±261.58)	116.8 (±203.27)	89.3 (±183.32)	119.13 (±239.31)	104.17 (±208.75)	107.23 (±234.63)	122.3 (±230.95)	135.05 (±276.11)
**CHILDREN (<2 YEARS)**												
Brazil	268.16 (±261.87)	245.38 (±252.82)	308.68 (±321.55)	231.17 (±259.13)	230.85 (±256.40)	207.63 (±234.25)	206.32 (±246.08)	184.72 (±206.53)	183.23 (±218.43)	170.68 (±209.93)	178.86 (±208.04)	175.94 (±205.68)
North	355.87 (±337.81)	344.07 (±332.11)	422.77 (±388.52)	345.55 (±316.94)	293.85 (±282.95)	280.62 (±268.87)	273.01 (±270.11)	222.57 (±213.91)	226.19 (±225.41)	201.75 (±207.89)	206.24 (±205.55)	209.65 (±204.08)
Northeast	278.07 (±263.68)	271.30 (±267.69)	366.06 (±367.31)	274.67 (±293.05)	253.40 (±285.54)	235.84 (±265.48)	223.55 (±257.97)	197.36 (±223.02)	196.26 (±250.16)	190.43 (±249.81)	191.72 (±242.95)	182.39 (±223.34)
Southeast	200.87 (±204.65)	179.57 (±189.70)	227.21 (±257.72)	172.88 (±212.07)	193.34 (±223.93)	162.44 (±201.74)	173.08 (±243.65)	155.50 (±191.23)	152.60 (±183.92)	142.44 (±183.92)	153.26 (±176.43)	152.84 (±197.18)
South	304.48 (±267.04)	259.17 (±256.30)	289.02 (±279.49)	206.13 (±221.10)	229.95 (±240.68)	200.42 (±199.81)	201.60 (±211.67)	193.31 (±198.35)	193.14 (±216.33)	174.27 (±189.34)	187.84 (±193.69)	187.01 (±199.90)
Midwest	297.18 (±286.38)	253.52 (±239.78)	322.95 (±289.57)	228.53 (±236.57)	219.86 (±237.78)	210.18 (±227.53)	208.54 (±247.48)	182.96 (±194.38)	176.63 (±184.12)	157.70 (±164.08)	172.05 (±195.82)	174.21 (±170.11)

Sd, standard deviation

**Fig 1:**
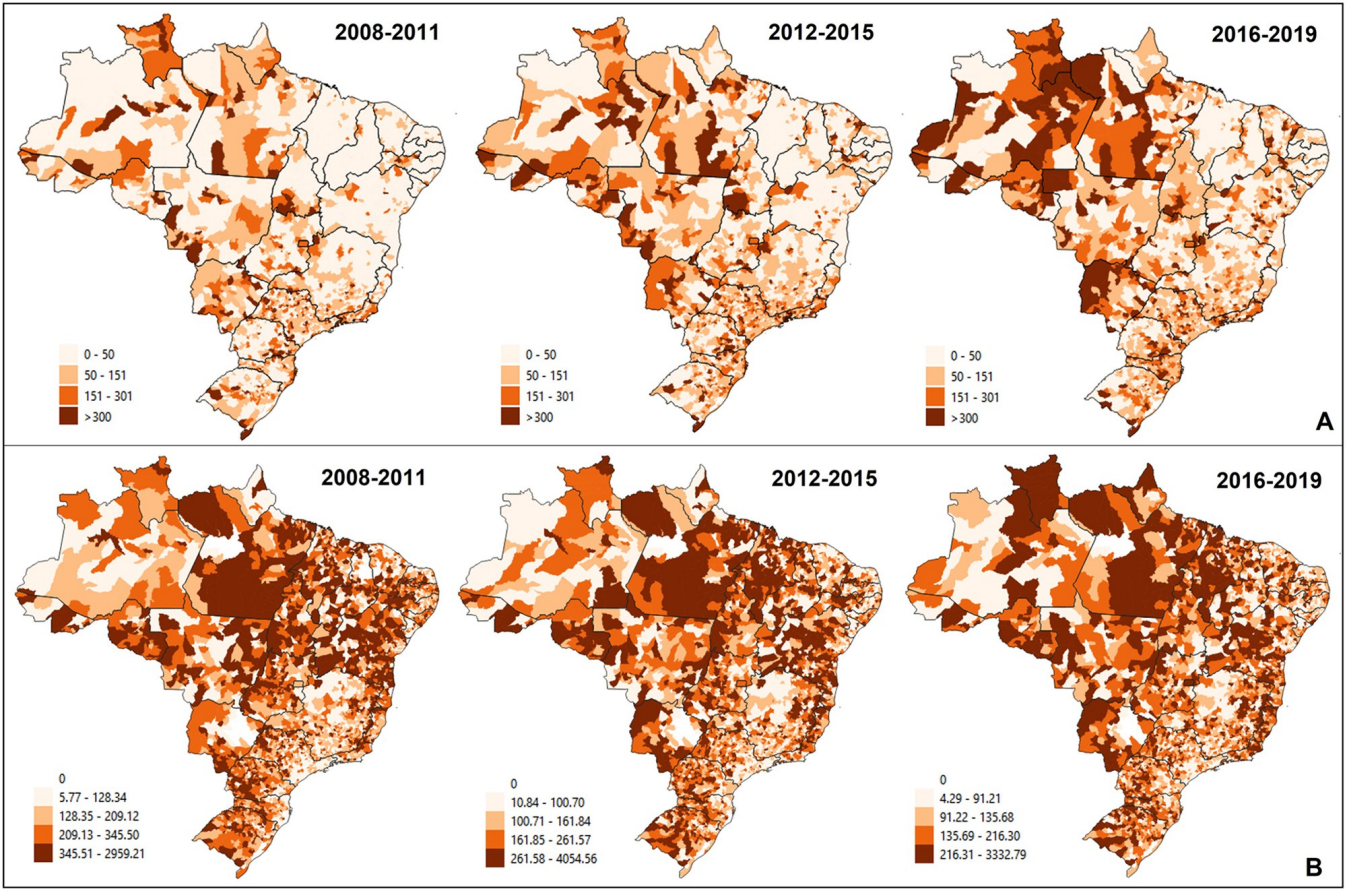
Smoothed PCSC rate in the first thousand days. Brazil, 2008–2019. (A) PCSC rate in women due to causes related to related to prenatal care and childbirth. (B) PCSC rate in children under two years of age.

Higher rates were found in municipalities in the North and Midwest for the women-related component (Fig 1A). Higher rates were found in municipalities in the North, Northeast, Midwest, and scattered areas in other regions concerning children under two years of age. Despite the general improvement in this indicator, some areas have deteriorated over the four-year periods evaluated, as is the case in the state of Roraima (Fig 1B).

**Fig 2:**
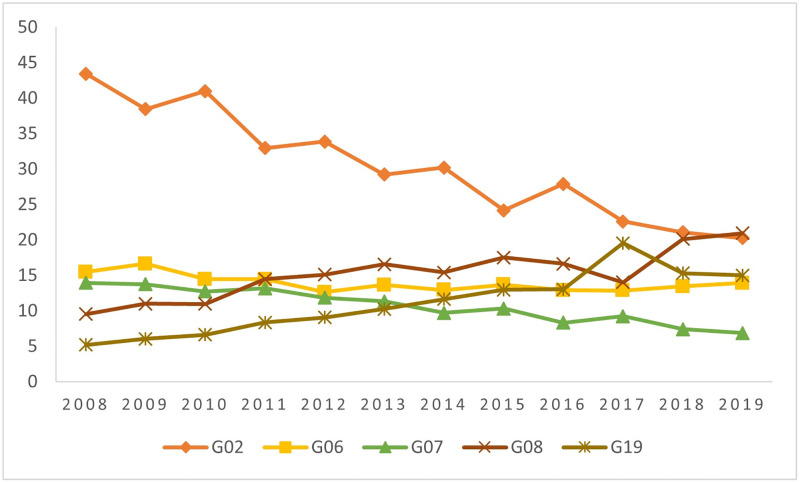
Proportion of ACSC by groups of causes, considering the five most frequent causes. Brazil, 2008–2019. G02—Infectious gastroenteritis and complications; G06—Bacterial pneumonia; G07—Asthma; G08—Lung diseases; G19—Diseases related to prenatal care and childbirth.

The causes of PCSC in this population group have changed over the years. In 2008, 43.4% of PCSC were caused by diseases in the “Infectious Gastroenteritis and Complications (G2)” group, which decreased over the years, showing half of this percentage in 2019. On the other hand, diseases related to prenatal care and childbirth (G19), which comprised 5% of PCSC at the onset of the period, showed a significant increase over the years, reaching 15% of PCSC in 2019 (Fig 2).

**Fig 3:**
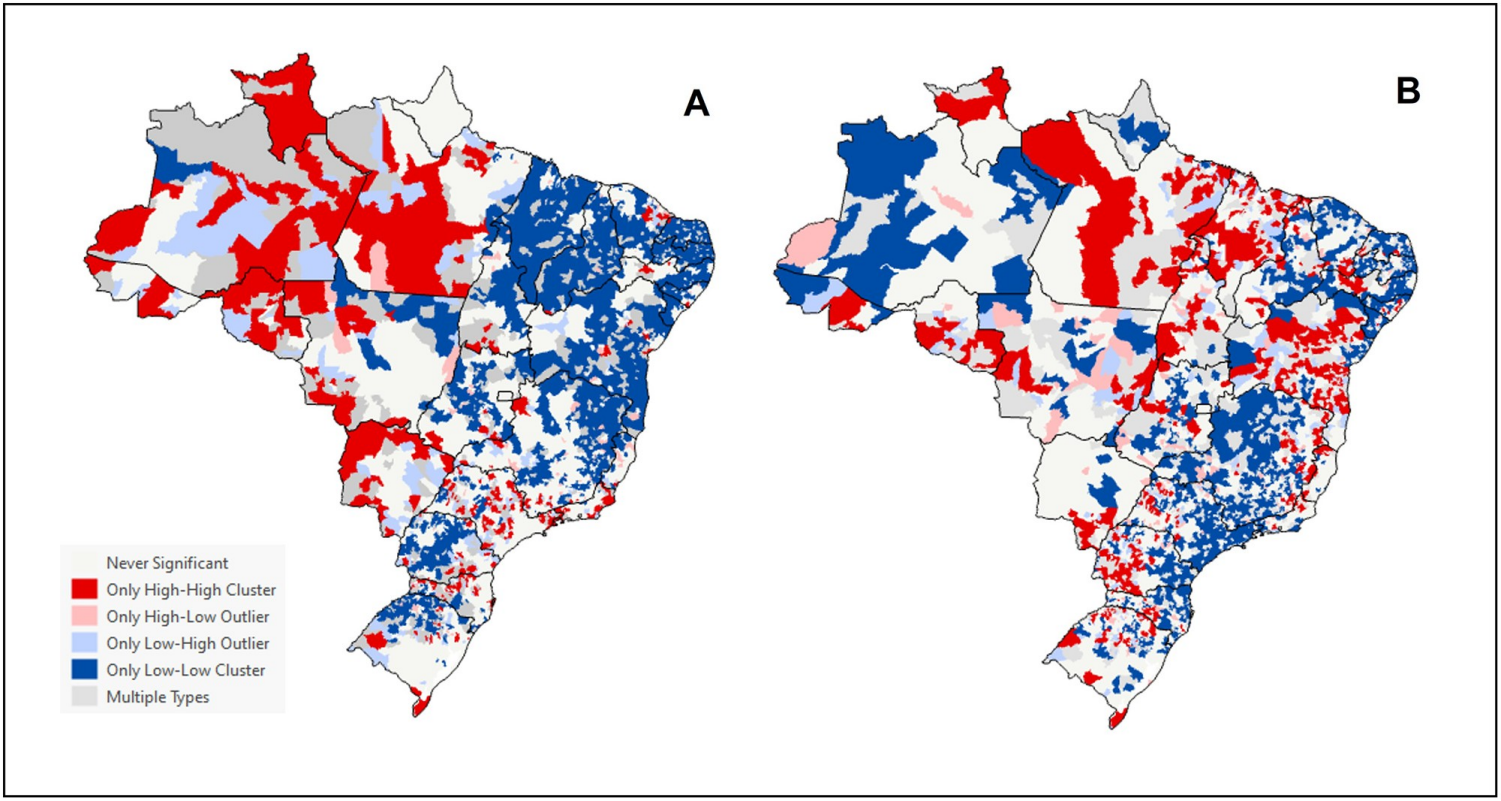
Local outlier analysis of PCSC related to prenatal care and childbirth and in children under two years old. Brazil, 2008–2019. (A) PCSC in women, for causes related to related to prenatal care and childbirth. (B) PCSC in children under two years of age.

In women, high-risk clusters for PCSC (High-High pattern) were found in the North and Midwest regions (Fig 3A). In children under two years old, high-risk clusters for PCSC were also found in these regions and the Northeast region, even with the general declining trend (Fig 3B).

**Fig 4:**
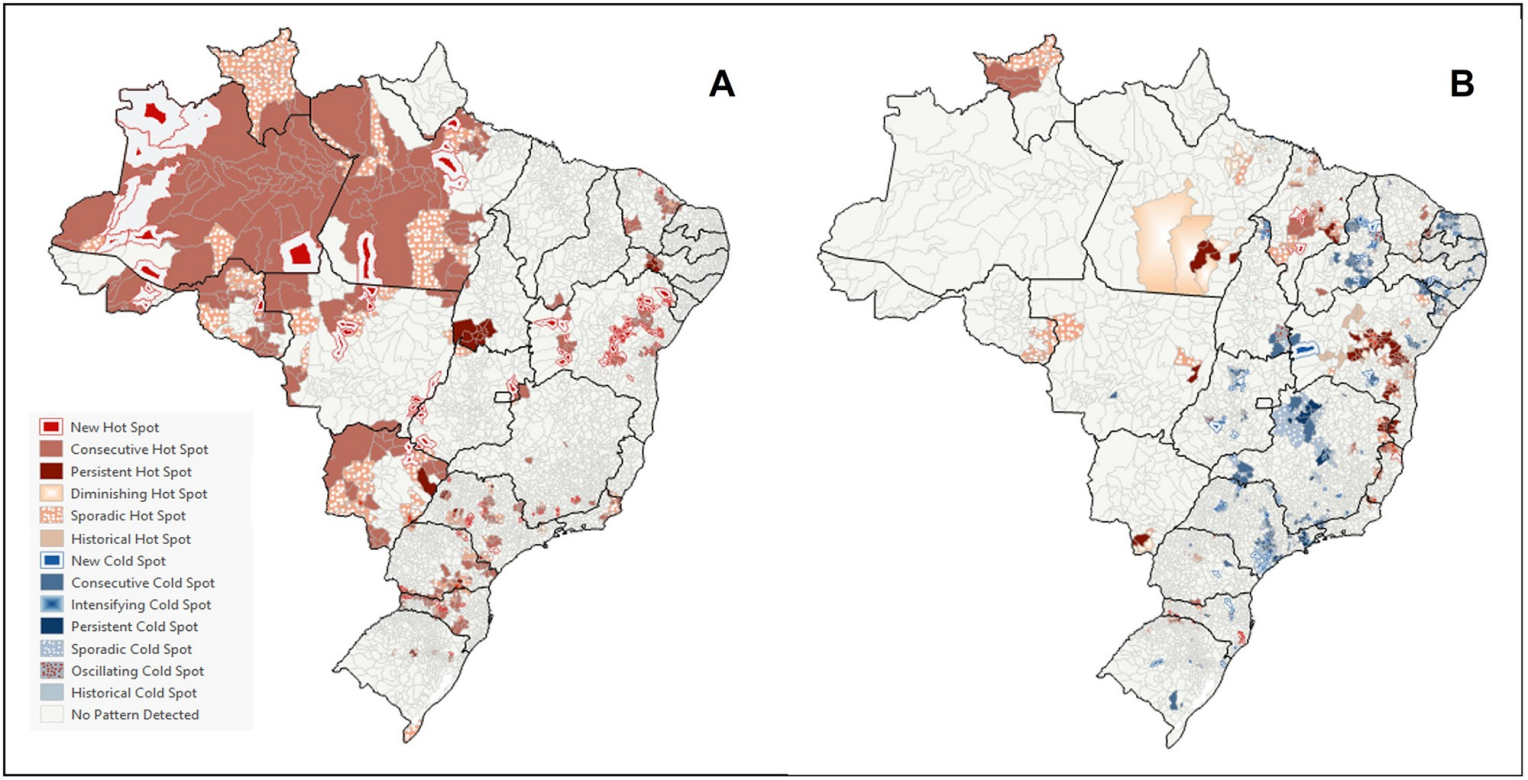
Emerging hotspot analysis, from a space-time cube, of PCSC related to prenatal care and childbirth and children under two years old. Brazil, 2008–2019. (A) PCSC in women, for causes related to prenatal care and childbirth. (B) PCSC in children under two years of age.

We observed an increase in PCSC rates in women due to causes related to prenatal care and childbirth, seen from the presence of hotspots in all regions of the country, especially in the North and Midwest and scattered areas in other regions. The predominant clusters, spatially in the North region of the country, were those of the consecutive hotspot type, representing locations that emerged as hotspots at the end of the time series; and sporadic hotspot (locations where they emerged as hotspots in less than 90% of time intervals, but never as cold spots). Besides these patterns, persistent hotspot clusters were also identified in small areas of Tocantins and Mato Grosso do Sul, indicating locations with high rates in more than 90% of the time series and new hotspot clusters, indicating locations that emerge as hotspots only at the end of the time series (Fig 4A).

Concerning PCSC rates in children under two years of age, we identified spatiotemporal clusters with a general declining trend in areas distributed throughout the country’s regions. However, clusters of the following types were identified in some municipalities in the North and Northeast regions: i) **persistent hotspot**, which represents locations with hotspots in more than 90% of the time series; ii) **diminishing hotspot**, which represents the locations that are hotspots along the time series, but show a reduction in their value; iii) **sporadic hotspot**, locations where they emerged as a hotspot in less than 90% of time intervals, but never as a cold spot; and vi) **consecutive hot spot**, representing locations that emerged as hot spots at the end of the time series (Fig 4B).

**Fig 5:**
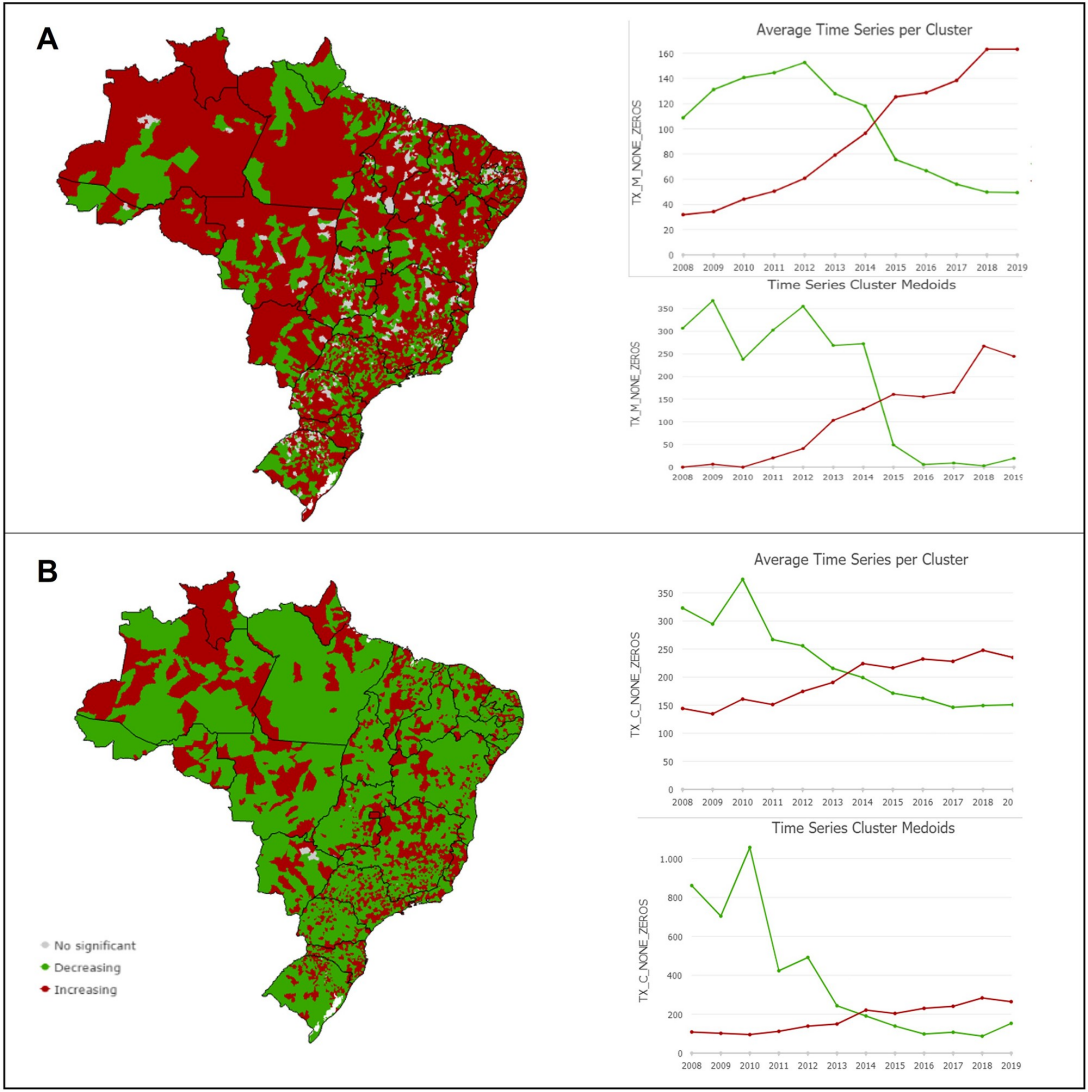
Time-series clustering of PCSC over the first thousand days. (A) Spatiotemporal clusters of PCSC rates in women due to causes related to prenatal care and childbirth. (B) Spatiotemporal clusters of PCSC rates in children under two years old.

Regarding the temporal trend, considering the spatial component, the municipalities were categorized into three groups: increasing trend, decreasing trend, and non-significant trend. In women, clusters with an increasing PCSC trend (F = 4.4; p = 0.004) were observed in most Brazilian municipalities (n = 3,242). The municipalities with a declining PCSC trend among women showed a decreasing overall mean rate from 2012 (Fig 5A). The clusters showed a downswing in children under two years of age (F = -3.77; p = 0.002) in this indicator in most of the country (n = 3,869 municipalities), with a persistent growing trend in specific areas scattered throughout the territory (Fig 5B).

## Discussion

In Brazil, PCSC rates in women and children in the first 1000 days of life from 2008 to 2019 behaved differently, with a more favorable setting for children under two years old than women. Additionally, spatiotemporal analysis identified areas of the country facing challenges to avoid PCSC in the first thousand days of life.

While it is important to point out the existence of other determinants in the reduction of PCSC in Brazil, studies relate the decline of PCSC in children, above all, to the expanding PHC coverage [4–6, 20]. PCSC downswing trends in children affect the declining infant mortality rates in the country, which dropped, especially in the first year of life [9]. In this sense, it is also worth highlighting PHC’s role in increasing the number of children who are exclusively breastfed up to six months of life [9], a protective factor for several childhood diseases [21].

Also, in this scope, noteworthy is the implementation of the Brazilian National Program for Improving Access and Quality of Primary Care (PMAQ), a pay-for-performance program implemented in 2011 [22]. The adherence of municipalities to the PMAQ, considering the three cycles of the program (2011–2018), showed positive effects in reducing PCSC, especially in the under-5 age group [23].

Besides PHC’s direct actions, the *Bolsa Família* conditional cash transfer program encouraged access to health services and food, improving nutritional status and reducing child mortality rates. In the nutritional field, we have seen a declining prevalence of height and weight deficits for age in the last two decades, equating some regions, such as the Northeast to the Southeast regions [9]. These associated factors may be related to lower PCSC in children under two years of age.

Despite the declining trend, we still identified sensitive areas in the country, and high PCSC rates persist in children under two years of age, which may be because that location and access to health services are determining factors for improving maternal and neonatal care. Thus, the availability of health services alone does not ensure the prevention of hospital admissions [24].

Socioeconomic and demographic factors can also modify the behavior of this indicator. A Brazilian study evaluating PCSC in children under the age of five points to inequalities in this indicator’s rates when evaluating the ethnicity/skin color component, which indicates that indigenous children had higher admission rates in the North and Midwest regions [25].

In contrast to the improvements identified in children under two years of age, we noted a growing trend over the years when assessing PCSC rates in pregnant women/puerperae. Other studies that only evaluated the component of PCSC related to childbirth and postpartum showed a slight reduction [7] and even an increase in hospital admissions related to this group of conditions [26].

The maternal mortality ratio showed a slight decrease in the last decade in Brazil, being far from reaching adequate numbers. Perinatal conditions are the leading group of causes of death in Brazil and represent problems related to the quality of prenatal care (attribution of the PHC), but are also related to the access and quality of hospital care at the time of delivery and childbirth [9].

The North, Midwest, and Northeast areas evidenced the worst settings for PCSC related to prenatal care and delivery. Despite the general improvement in indicators related to prenatal care [27], we still find some differences in the services provided by the PHC at prenatal care, where the proportion of women who had six or more visits during pregnancy is higher among whites, with higher-income, and living in the South region. In contrast, lower-income women receive less guidance during prenatal care, especially in the Midwest and North regions [28].

Also, in this sense, a study that evaluated the quality of prenatal care carried out by PHC teams in Brazil points out that the North region had a lower prevalence of PHC facilities (UBS) with an adequate structure, while the South and Southeast regions had better structure, adequacy of test requests, and users receiving guidance [29]. These findings point to an improved prenatal care, but with the same differences related to the regions of the country, besides gaps related to the size of the municipality, population size, and HDI, and the structure was better in small municipalities with more extensive PHC coverage and the request for tests was more prevalent in larger municipalities with lower PHC coverage [29].

Our search efforts could not find other studies evaluating PCSC in the 1000 days in Brazil. Also, studies including parts of this population (pregnant women, puerperae, or children) were conducted using only local data before the period considered in our study and without considering the entire national territory.

As limitations of this work are the secondary data, which can lead to inaccurate and inconsistent information. In this sense, the available data did not allow us to identify PCSC for causes other than those indicated in the group "Diseases related to prenatal care and delivery", which can lead us to underestimate the PCSC rates in the group of pregnant women and puerperae. The ecological fallacy needs to be taken into account, so it is not possible to make inferences to the individual level. Another limitation is the non-inclusion of confounders on the analyses. Although our proposal has included spatial weightings of the indicators, we understand that other variables could help to explain the spatial distribution found in the study. So, future studies can further investigate the causes of PCSC in women in this period of life, including the role of the income level, the access to primary care services, and the availability of hospital beds in municipalities on the PCSC.

We can conclude that there is a reduction in Brazilian PCSC rates in children under two years of age. However, significant PCSC rates persist in pregnant women and puerperae, increasing over the years, indicating problems in accessing the health system or its performance. In this sense, the identification of sensitive areas for PCSC in the first thousand days can lead to local epidemiological investigations and guide managers’ actions in search of interventions in this reality, with a declining inequality in the distribution, access, and quality of health services provided within the Brazilian PHC.

## References

[1] Alfradique ME, Bonolo P de F, Dourado I, Lima-Costa MF, Macinko J, Mendonça CS, et al. Internações por condições sensíveis à atenção primária: a construção da lista brasileira como ferramenta para medir o desempenho do sistema de saúde (Projeto ICSAP—Brasil). Cad Saude Publica. 2009;25: 1337–49. Available at: http://www.ncbi.nlm.nih.gov/pubmed/19503964 doi: 10.1590/s0102-311x2009000600016 19503964

[2] Pinto LF, Giovanella L. Do Programa à Estratégia Saúde da Família: expansão do acesso e redução das internações por condições sensíveis à atenção básica (ICSAB). Cien Saude Colet. 2018;23: 1903–1914. doi: 10.1590/1413-81232018236.05592018 29972498

[3] de Q Costa L, Pinto EP, da Silva MGC, de Q Costa L, Pinto EP, da Silva MGC. Tendência temporal das Internações por Condições Sensíveis à Atenção Primária em crianças menores de cinco anos de idade no Ceará, 2000 a 2012. Epidemiol e Serviços Saúde. 2017;26: 51–60. 10.5123/S1679-49742017000100006 28226008

[4] Pazó RG, Frauches DDO, Molina MDCB, Cade NV. Panorama das internações por condições sensíveis à atenção primária no Espírito Santo, Brasil, 2000 a 2014. Rev Bras Med Família e Comunidade. 2017;12: 1–12. doi: 10.5712/rbmfc12(39)1546

[5] Carvalho SC, Mota E, Dourado I, Aquino R, Teles C, Medina MG. Hospitalizations of children due to primary health care sensitive conditions in Pernambuco State, Northeast Brazil. Cad Saude Publica. 2015;31: 744–754. doi: 10.1590/0102-311x00069014 25945984

[6] Barreto JOM. Estratégia Saúde da Família e internações hospitalares em menores de 5 anos no Piauí, Brasil The Family Health Strategy and hospital admissions of children under fi ve years in Piauí State, Brazil. Cad Saúde Pública, Rio Janeiro. 2012;28: 515–526. 10.1590/s0102-311x2012000300012 22415184

[7] Maciel AG, Diniz FJL de S, Caldeira AP. Impacto da Estratégia Saúde da Família sobre o perfil de morbidade hospitalar em Minas Gerais. Saúde em Debate. 2014;38: 319–330.

[8] de S Mendonça S, de Albuquerque EC. Perfil das internações por condições sensíveis à atenção primária em Pernambuco, 2008 a 2012. Epidemiol e Serviços Saúde. 2014;23: 277–286.

[9] Leal M do C, Szwarcwald CL, Almeida PVB, Aquino EML, Barreto ML, Barros F, et al. Saúde reprodutiva, materna, neonatal e infantil nos 30 anos do Sistema Único de Saúde (SUS). Cien Saude Colet. 2018;23: 1915–1928. doi: 10.1590/1413-81232018236.03942018 29972499

[10] Rosano A, Loha CA, Falvo R, Van Der Zee J, Ricciardi W, Guasticchi G, et al. The relationship between avoidable hospitalization and accessibility to primary care: a systematic review. Eur Public Heal. 2012;23: 356–360. doi: 10.1093/eurpub/cks053 22645236

[11] Alfradique ME, Bonolo P de F, Dourado I, Lima-Costa MF, Macinko J, Mendonça CS, et al. Ambulatory care sensitive hospitalizations: Elaboration of Brazilian list as a tool for measuring health system performance (project ICSAP—Brazil). Cad Saude Publica. 2009;25: 1337–1349. doi: 10.1590/s0102-311x2009000600016 19503964

[12] Cusick SE, Georgieff MK. The Role of Nutrition in Brain Development: The Golden Opportunity of the “First 1000 Days.” J Pediatr. 2016;175: 16–21. doi: 10.1016/j.jpeds.2016.05.013 27266965 PMC4981537

[13] Grantham-McGregor S, Cheung YB, Cueto S, Glewwe P, Richter L, Strupp B. Developmental potential in the first 5 years for children in developing countries. Lancet. 2007;369: 60–70. doi: 10.1016/S0140-6736(07)60032-4 17208643 PMC2270351

[14] IBGE. Estatísticas. 2020 [cited 23 October 2020]. In: IBGE [Internet] https://www.ibge.gov.br/estatisticas/sociais/saude.html

[15] BRASIL. Portaria GM/MS n°. 2.848 de 06 de novembro de 2007. Brasília: Diário Oficial da União; 2007.

[16] EMBRAPA. Análise espacial de dados demográficos. Brasília; 2004.

[17] Anselin L. Local Indicators of Spatial Association—LISA. Geogr Anal. 1995;27: 93–115. doi: 10.1111/J.1538-4632.1995.TB00338.X

[18] ESRI. How Emerging Hot Spot Analysis works. In: ArcGIS Pro 2.7 [Internet]. [cited 24 Jul 2021]. https://pro.arcgis.com/en/pro-app/latest/tool-reference/space-time-pattern-mining/an-overview-of-the-space-time-pattern-mining-toolbox.htm

[19] ESRI. How Time Series Clustering works. In: ArcGIS Pro 2.7 [Internet]. https://pro.arcgis.com/en/pro-app/latest/tool-reference/space-time-pattern-mining/learnmoretimeseriesclustering.htm

[20] Ceccon RF, Meneghel SN, Viecili PRN. Internações por condições sensíveis à atenção primária e ampliação da Saúde da Família no Brasil: um estudo ecológico. Rev Bras Epidemiol. 2014;17: 968–977. 25388495 10.1590/1809-4503201400040014

[21] Victora CG, Bahl R, Barros AJD, França GVA, Horton S, Krasevec J, et al. Breastfeeding in the 21st century: Epidemiology, mechanisms, and lifelong effect. Lancet. 2016;387: 475–490. doi: 10.1016/S0140-6736(15)01024-7 26869575

[22] Macinko J, Harris MJ, Rocha MG. Brazil’s national program for improving primary care access and quality (PMAQ) fulfilling the potential of the world’s largest payment-for-performance system in primary care. J Ambul Care Manage. 2017;40: S4–S11. doi: 10.1097/JAC.0000000000000189 28252498 PMC5338882

[23] Russo LX, Powell-Jackson T, Maia Barreto JO, Borghi J, Kovacs R, Gurgel Junior GD, et al. Pay for performance in primary care: The contribution of the Programme for Improving Access and Quality of Primary Care (PMAQ) on avoidable hospitalisations in Brazil, 2009–2018. BMJ Glob Heal. 2021;6: 1–9. doi: 10.1136/bmjgh-2021-005429 34244203 PMC8273460

[24] Ebener S, Guerra-Arias M, Campbell J, Tatem AJ, Moran AC, Amoako Johnson F, et al. The geography of maternal and newborn health: The state of the art. Int J Health Geogr. 2015;14. doi: 10.1186/s12942-015-0012-x 26014352 PMC4453214

[25] Farias YN, Leite IDC, Siqueira MAMT De, Cardoso AM. Ethnic and racial inequalities in hospital admissions due to avoidable causes in under-five Brazilian children, 2009–2014. Cad Saude Publica. 2019;35: 1–14. doi: 10.1590/0102-311x00001019 31433026

[26] de S Mendonça S, de Albuquerque EC. Investigação epidemiológica dos óbitos notificados tendo como causa básica a hanseníase, ocorridos em Fortaleza, Ceará, 2006–2011. Epidemiol e Serviços Saúde. 2014;23: 277–286. doi: 10.5123/S1679-49742014000200009

[27] França GVA, Restrepo-Méndez MC, Maia MFS, Victora CG, Barros AJD. Coverage and equity in reproductive and maternal health interventions in Brazil: Impressive progress following the implementation of the Unified Health System. Int J Equity Health. 2016;15: 1–12. 27852276 10.1186/s12939-016-0445-2 PMC5112713

[28] Tomasi E, Fernandes PAA, Fischer T, Siqueira FCV, da Silveira DS, Thumé E, et al. Qualidade da atenção pré-natal na rede básica de saúde do Brasil: Indicadores e desigualdades sociais. Cad Saude Publica. 2017;33: 1–11. doi: 10.1590/0102-311X00195815 28380149

[29] Neves RG, Flores-Quispe MDP, Facchini LA, Fassa AG, Tomasi E. Pré-natal no Brasil: estudo transversal do Programa de Melhoria do Acesso e da Qualidade da Atenção Básica, 2014. Epidemiol e Serv saude Rev do Sist Unico Saude do Bras. 2020;29: e2019019. doi: 10.5123/S1679-49742020000100008 32074198

